# Biomechanical analysis of the correlation between mid-shaft atypical femoral fracture (AFF) and axial varus deformation

**DOI:** 10.1186/s13018-022-03060-1

**Published:** 2022-03-15

**Authors:** Mathieu Severyns, Dalila Belaid, Kevin Aubert, Ali Bouchoucha, Arnaud Germaneau, Tanguy Vendeuvre

**Affiliations:** 1 Department of Orthopaedic Surgery and Traumatology, Hôpital Pierre Zobda Quitman, University Hospital, 97261 Fort-de-France Cedex, Martinique France; 2 Institute Pprime UPR 3346, CNRS – University of Poitiers – ISAE-ENSMA, Poitiers, France; 3 Department of Mechanical Engineering, Faculty of Technology Sciences, University of Mentouri Brothers Constantine, Ain-El-Bey Way, P.O Box 325, 25017 Constantine, Algeria

**Keywords:** Atypical femoral fracture, Finite elements, Fracture risk indicator

## Abstract

**Background:**

Atypical femoral fractures (AFF) are diaphyseal fractures of the elderly that occur at the end of a minor trauma. The objective of this biomechanical study, using finite element modelling, was to evaluate the variations of the femoral diaphysis fracture indicator according to the variations of the mechanical axis of the lower limb, which can explain all the different atypical fracture types identified in the literature.

**Methods:**

In order to measure variations in stress and risk factors for fracture of the femoral diaphysis, the distal end of the femur was constrained in all degrees of freedom. An axial compression load was applied to the femoral head to digitally simulate the bipodal support configuration in neutral position as well as in different axial positions in varus/valgus (− 10°/10°).

**Results:**

The maximum stress value of Von Mises was twice as high (17.96 ± 4.87 MPa) at a varus angle of − 10^°^ as in the neutral position. The fracture risk indicator of the femoral diaphysis varies proportionally with the absolute value of the steering angle. However, the largest simulated varus deformation (− 10°) found a higher risk of diaphysis fracture indicator than in valgus (10°).

**Conclusions:**

Variations in the mechanical axis of the lower limb influence the stress distribution at the femur diaphysis and consequently increase the risk of AFF. The axial deformation in varus is particularly at risk of AFF. The combination of axial deformation stresses and bone fragility consequently contribute to the creation of an environment favorable to the development of AFF.

*Trial registration*: ‘retrospectively registered’.

## Introduction

Atypical femoral fractures (AFF) are diaphyseal fractures of the elderly that occur as a result of minor trauma and may extend from the small trochanter to the supra-condylian metaphyso-diaphyseal junction [1]. Strict diagnostic criteria have recently been developed by the American Society for Bone and Mineral Research (ASBMR) [2]. Four of the five major criteria must be met to make the diagnosis: (1) Minor trauma such as a fall from a height or absence of identified trauma, (2) Oblique or transverse fracture involving the external cortical, (3) Complete quadri-cortical fracture with a medial scale or incomplete fracture of interest to the lateral cortical, (4) Small or non-comminutive fracture, and (5) Localized thinning of the periosteum or endostus of the external cortical. The concepts of iatrogenia (biphosphonates, glucocorticoids, proton pump inhibitors [3–5]) or comorbidities (rheumatoid arthritis, chronic kidney failure [6, 7]) are now reduced to minor criteria. Indeed, several scientific studies [8–11] have supported the mechanical theory that these atypical fractures are linked to an abnormal distribution of stresses on the lateral cortical area of the femur. Two major types of AFF now appear to be emerging: medial-diaphyseal fatigue fracture on curved femur and sub-trochanteric fracture [12]. The hypothesis of this work was that the higher the axial varus deformation of the lower limb the higher probability of AFF occuring. The objective of this biomechanical study, using finite element modelling, was therefore to evaluate the variations of the femoral diaphysis fracture indicator according to the variations of the mechanical axis of the lower limb, which can explain all the different atypical fracture types identified in the literature.

## Methods

The analysis of load distribution and the effect of lower limb alignment was made possible by finite element modelling constructed from whole human femur CT scans (*n* = *5*). The epidemiological and radiological characteristics of patients are summarized in Table 1.

**Table 1:** Epidemiological and radiographic characteristics of whole femurs modelled as finite elements

Femur subject	AA	BB	CC	DD	EE
Age (year)	73	71	88	65	60
Gender	M	M	M	F	F
Side	R	L	R	R	L
Length (mm)	437.77	444.44	390.81	356.36	384.38
Neck-shaft angle (deg)	128.41	130.93	134.02	125.13	123.42
Hip-knee shaft angle (deg)	5.46	4.34	5.24	7.27	6.38

### Finite element modeling (FE)

The methodology used to generate the numerical models was to reconstruct the entire femur from the CT scan acquisition. The X-ray scan parameters for the samples were set to 120 kVp, 100 mAs, with 0.75 mm cross-sections and an image matrix of 512 × 512 pixels with a pixel size of 0.434 mm. Volume image segmentation and geometric model were performed using 3D SLICER software (Version 4.11, Kitware, France). To perform finite element modeling, the resulting model was imported and analyzed using Ansys® Workbench software (Version 2020R2, Ansys Inc, United States). Bone segment geometry was discretized using ten-node tetrahedral element (C3D10), and mesh sensitivity was analyzed for elements of different sizes, as recommended in the literature [10, 13, 14].

**Fig. 1:**
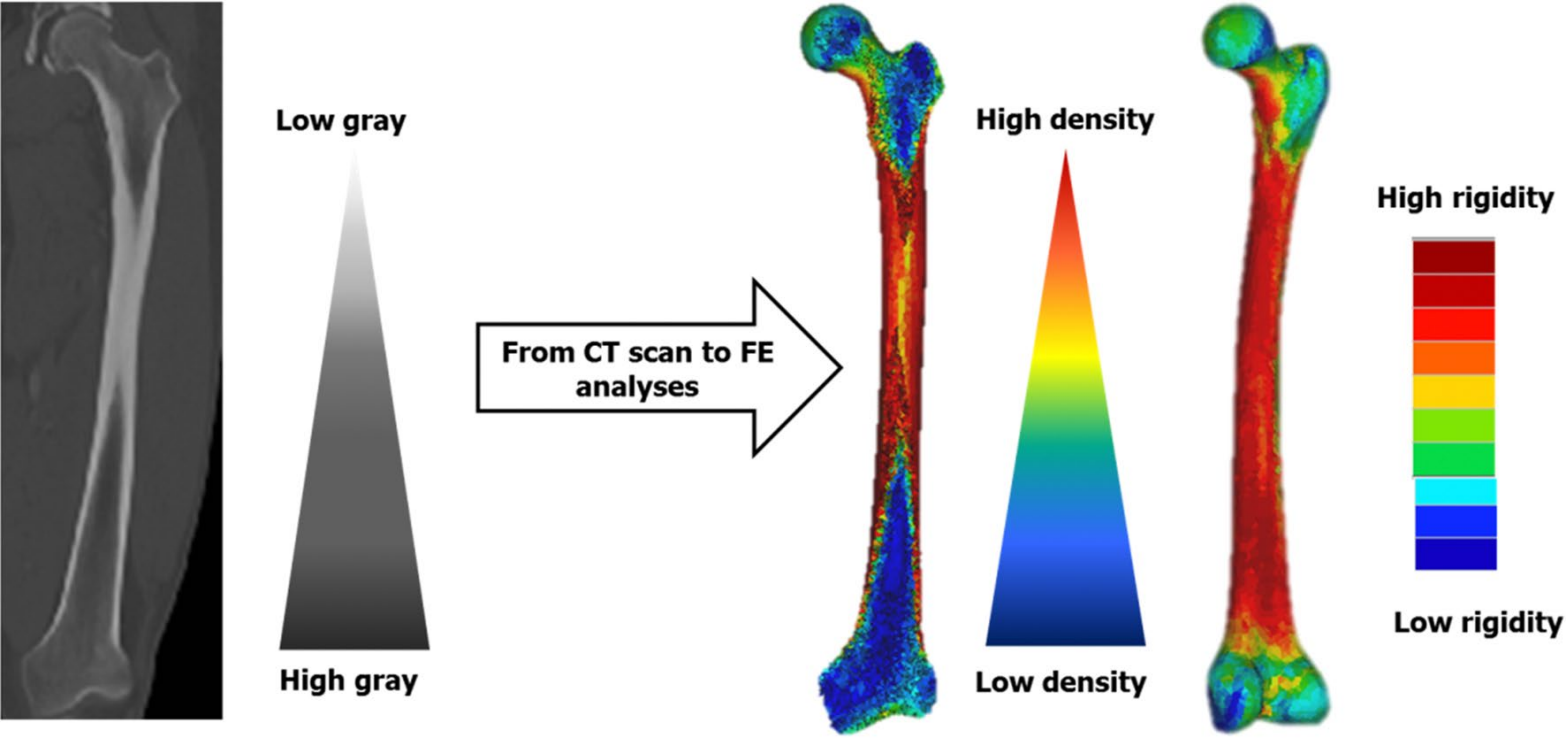
Femur bone density mapping (subject AA)

The model elasticity modulus have been assigned element by element. Bone is considered heterogeneous and isotropic. For all models, a Poisson coefficient of 0.3 was assigned [15]. The grayscale values from the CT scan data (Fig. 1) express the Hounsfield (HU) units that identify bone density from the relationship (1) below with $\rho_{{{\text{QCT}}}}$ as peripheric quantitative computed tomography [16]:

1 $$\rho_{{{\text{QCT}}}} \left( {{\text{g}}/{\text{cm}}^{3} } \right) = 0.007764 \times {\text{HU}} - 0.056148$$

In this work, the Young modulus of cortical and cancellous bones [17] allocated to each element was determined from the relationships below:

2 $$\rho_{{{\text{ash}}}} \left( {{\text{g}}/{\text{cm}}^{3} } \right) = 0.877 \times \rho_{{{\text{QCT}}}} + 0.0789$$

3 $$\rho_{{{\text{app}}}} \left( {{\text{g}}/{\text{cm}}^{3} } \right) = \rho_{{{\text{ash}}}} /0.6$$

4 $$E\left( {{\text{MPa}}} \right) = 6850 \times \rho_{{{\text{app}}}}^{1.49}$$

with $\rho_{{{\text{ash}}}}$ as ash density, $\rho_{{{\text{app}}}}$ as apparence density and E as elasticity module.

### Alignment axes, loading and boundary conditions

**Fig. 2:**
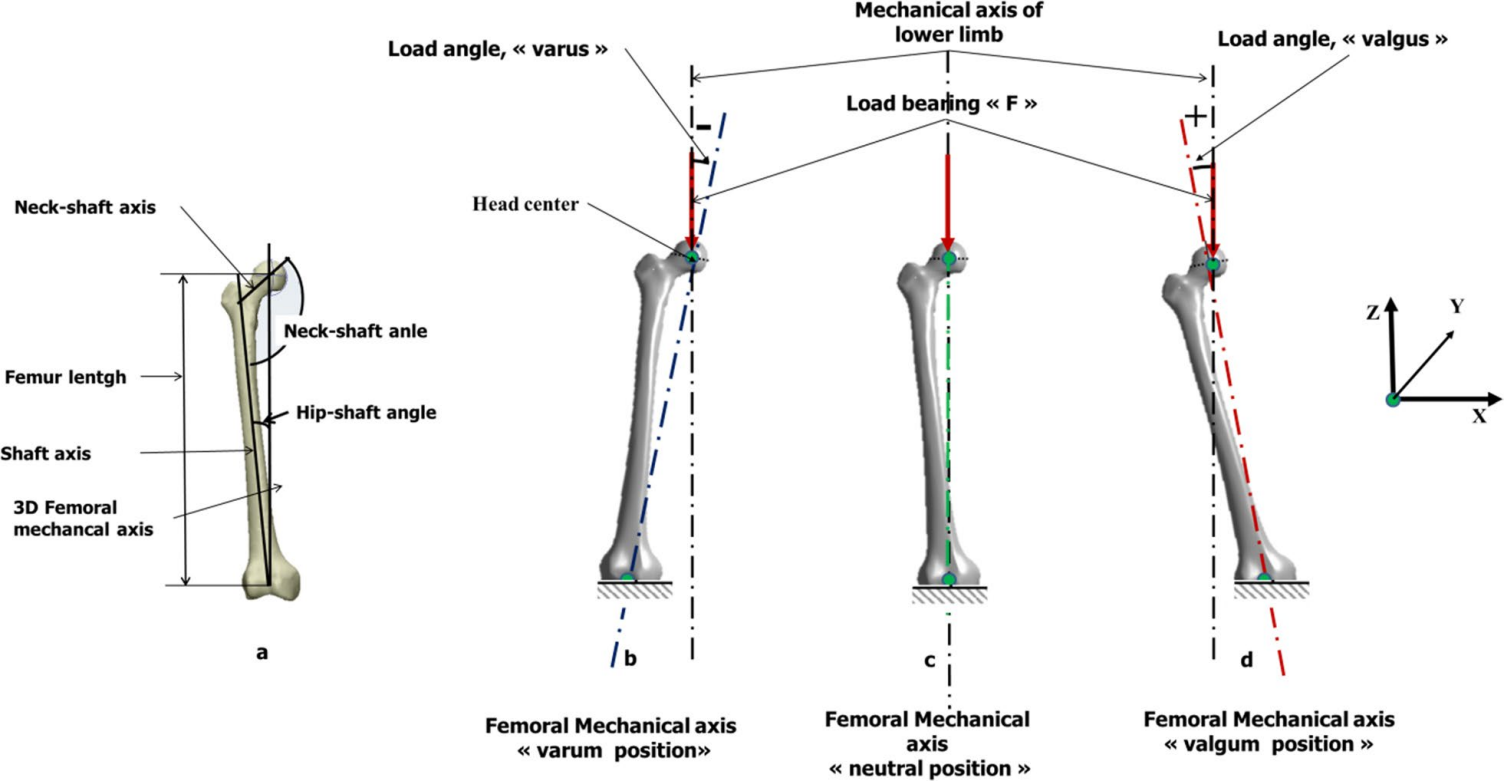
Femur alignment and Boundary Conditions. In the neutral position, the loading axis, femur mechanical axis and lower limb mechanical axis are vertical (angle 0°)

To determine the impact of lower limb alignment on stress amplitude and fracture risk factor, a load protocol corresponding to the standing position on both legs was modelled. In this scenario, in order to simulate the misalignment of the femur, it was necessary to identify the mechanical axes or loading axes. Reconstruction CT images were used to measure alignment [18]. The mechanical axis of the femur was considered a line connecting the center of the femur head and the center of the knee. In order to measure the resistance of the femur, the distal end of the femur was constrained in all degrees of freedom. An axial compression load corresponding to an average weight of 70 kg (686 N) was applied to the center of the femoral head in neutral position as well as in varus/valgus position by rotating the femur around the center of the femoral head clockwise or counterclockwise in relation to the frontal plane (Fig. 2).

### Post-treatments

Although the fracture force prediction and fracture site were based on the criteria of the maximum principal stress fracture theory, the fracture of a certain element has been defined as occurring when the maximum stress of the element exceeds its elastic limit. The atypical diaphyseal femur fracture risk indicator (FRI) was used for all load configurations, elucidating the effect of the anatomical variations of the femurs, their material properties and the mechanism of these fractures. The risk indicator for tensile fracture (TFRI) and femoral diaphysis compression (CFRI) was the ratio of ultimate (maximum) bone stress to limit stress and can be expressed as follows:

5 $${\text{CFRI}} = \frac{{\sigma_{{\text{min,ppal}}}^{C} }}{{\sigma_{{\text{y,ppl}}}^{C} }}$$

6 $${\text{TFRI}} = \frac{{\sigma_{{\text{max,ppal}}}^{T} }}{{\sigma_{{\text{y,ppl}}}^{T} }}$$

where $\sigma_{{\text{y,ppl}}}^{C}$ and $\sigma_{{\text{y,ppl}}}^{T}$ are respectively the stress limits in compressive and tensile strength;$\sigma_{{\text{max,ppal}}}^{T}$ and $\sigma_{{\text{min,ppal}}}^{C}$ are respectively the maximum and minimum main stresses.

After each load step, elements with the principal stress ($\sigma_{{{\text{ppl}}}}$) exceeding the yield stress ($\sigma_{{\text{Y,ppl}}}$) were “failed” by assigning a very small Young’s modulus (1 MPa). The relationships used between $\sigma_{{\text{y,ppl}}}$ t and $\rho_{{{\text{app}}}}$ proposed by (Kheirollahi, et al.) [19]were stated as:

7 $$\sigma_{y} = 116\rho_{{{\text{ash}}}}^{2.03} \left( {{\text{MPa}}} \right)$$

## Results

The alignment of the lower limb influences the distribution of stresses in the femoral diaphysis in a bipodal load configuration. Deformation due to axial alignment variations also affects the risk of fracture.

### Stress distribution

**Fig. 3:**
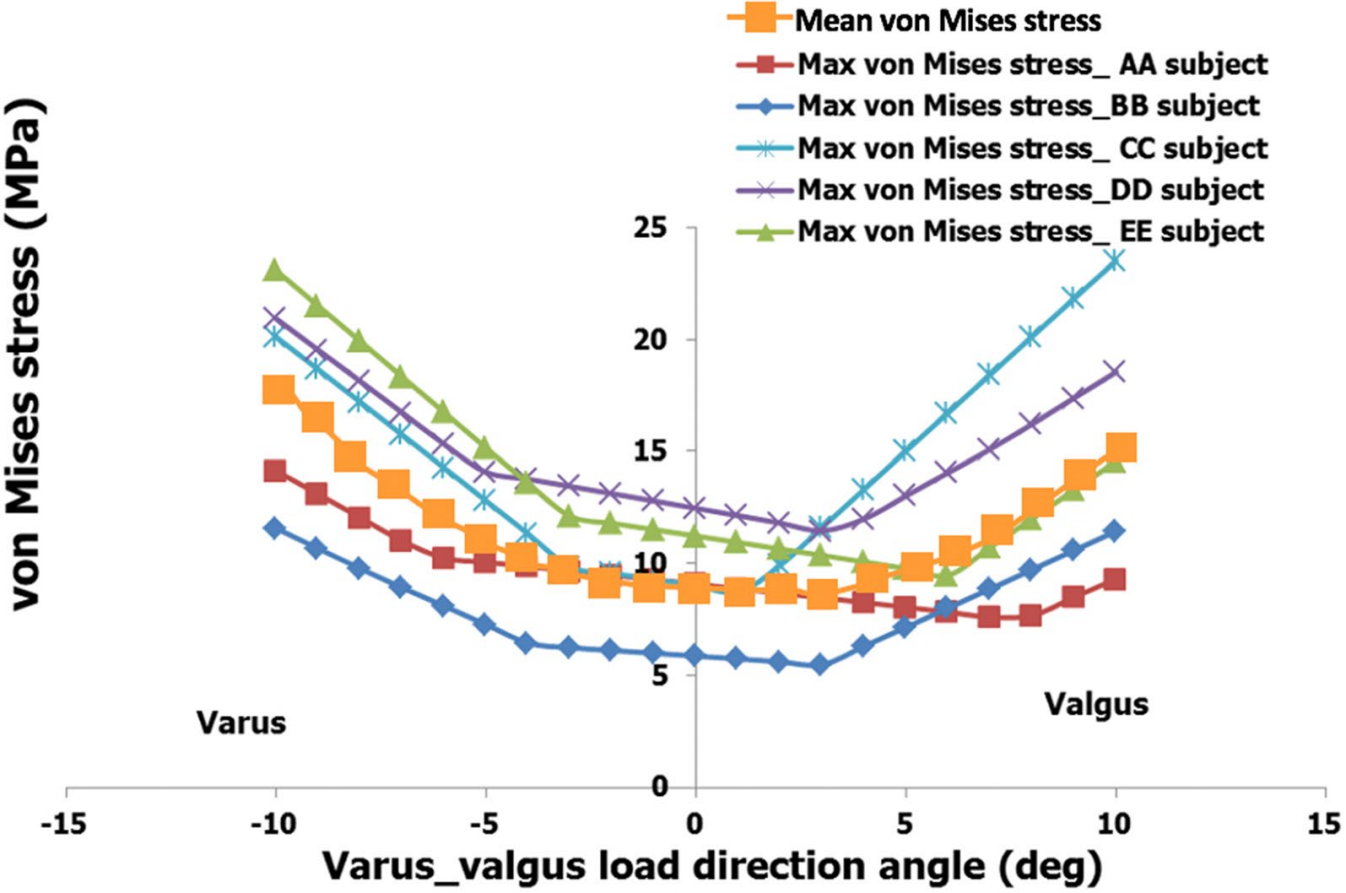
Maximum von Mises stress as a function of varus/valgus angle

Figure 3 shows the variation in von Mises equivalent stress for all subjects. The mean maximum stress value of von Mises was 9.53 MPa (SD = 2.52 MPa) for angle 0° ± while the mean maximum stress value of von Mises was 1.63 times higher (15.47 MPa, SD = 5.68 MPa) for angle 10° (valgus) and the stress value almost twice (17.96 MPa, SD = 4.87 MPa) higher for the angle − 10° (Table 2). There is also a clear increase in stress during varus deformation.

**Table 2:** The von Mises (MPa), (whole femur) maximum stress averages in all varus/valgus loads for the five subjects

Maximum von Stress Bets (MPa)																					
Angle(deg)	− 10	− 9	− 8	− 7	− 6	− 5	− 4	− 3	− 2	− 1	0	1	2	3	4	5	6	7	8	9	10
Range	11.57–23.06	10.67–21.49	9.77–19.91	8.92–18.32	8.098–16.73	7.27–15.14	6.444–13.76	6.23–13.44	6.115–13.12	5.99–12.79	5.86–12.47	5.74–12.13	5.61–11.80	5.48–11.61	6.30–13.31	7.15–15.02	7.83–16.72	7.61–18.42	7.67–20.12	8.49–21.81	9.31–23.50
Average	17.96	16.69	15.41	14.14	12.93	11.87	10.99	10.26	10.02	9.78	9.53	9.29	9.33	9.48	9.99	10.60	11.22	12.15	13.15	14.31	15.47
SD	4.87	4.58	4.30	3.99	3.62	3.20	3.02	2.75	2.67	2.60	2.52	2.45	2.37	2.56	2.81	3.33	3.97	4.51	5.03	5.35	5.68

**Fig. 4:**
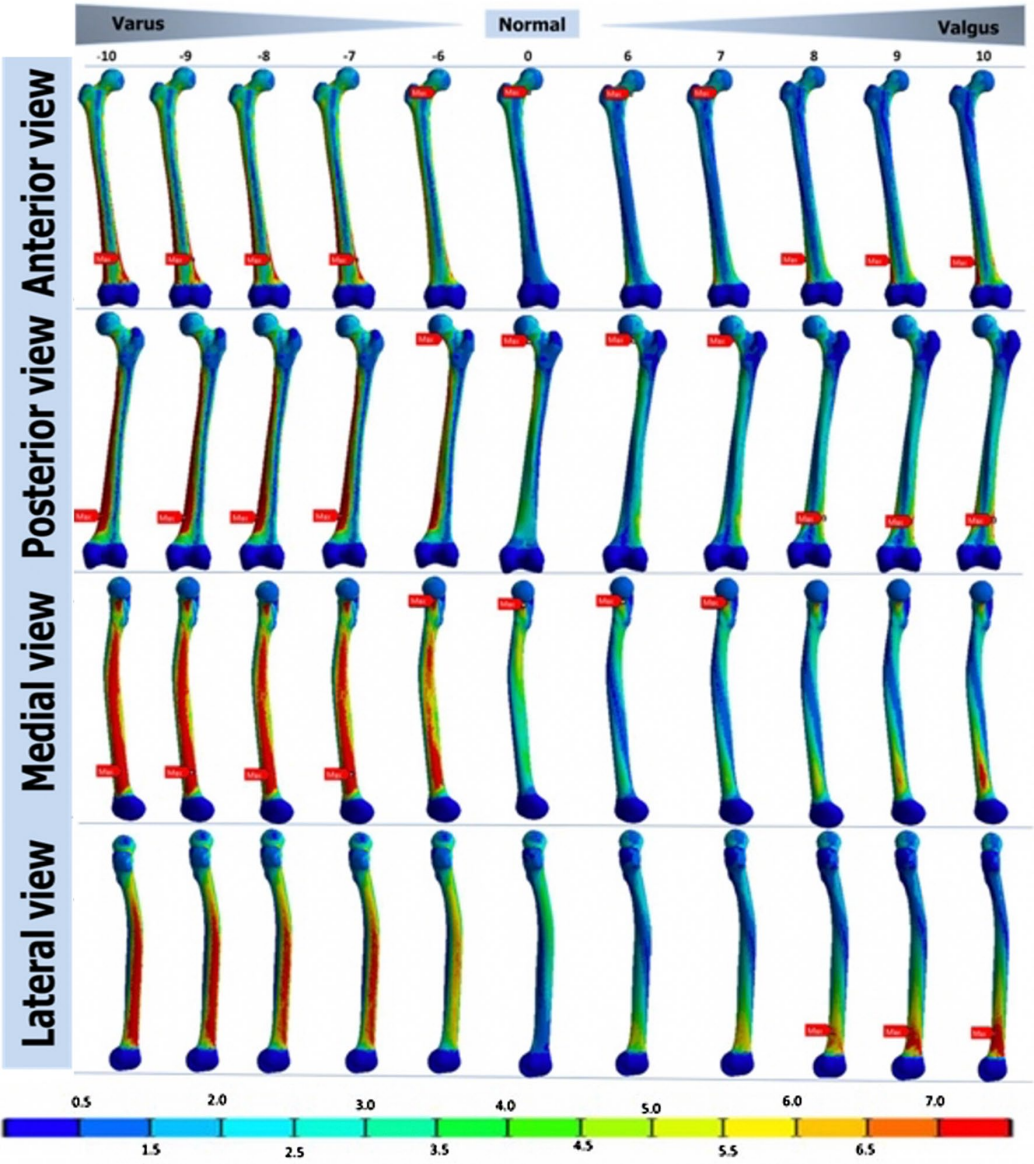
Maximum von Mises stresses of the diaphyseal area for the subject AA. Valgus/varus angles: between − 10^o^ and − 6° for valgus, between 6° and 10° for varus

Figure 4 shows stress distribution in the diaphysis region. The highest absolute stress value is located at 10° and − 10° angles and decreases as the angle approaches 0° (Table 3). Under the same load, (Table 3). Under the same load, and in the varus direction configuration, maximum main stress was localized at the lateral cortical femoral diaphysis, the minimum stress being localized at the medial cortical. On the contrary, under valgus stress, maximum main stress was localized at the antero-medial cortical level of the femoral diaphysis, minimum stress being localized at the lateral cortical. In addition, maximum stress was localized to the proximal diaphysis region in the neutral position and changed the localization to the distal diaphysis region at the valgus or varus angles. The stress values at the level of the femoral diaphysis evolved by varying the angle of varus or valgus.

**Table 3:** Means of the main strains of the femoral diaphysis in varus/valgus for the five subjects

Main stress (MPa)																					
Angle(deg)	− 10	− 9	− 8	− 7	− 6	− 5	− 4	− 3	− 2	− 1	0	1	2	3	4	5	6	7	8	9	10
*Minimum*																					
Range(abs	23.17–11.73	21.59–10.82	20.03–9.911	18.46–8.993	16.88–8.132	15.30–7.301	13.71–6.468	12.12–5.869	10.64–5.576	9.318–5.281	8.457–4.987	8.438–4.821	9.412–4.690	10.40–4.569	11.39–4.496	12.38–5.188	13.37–5.907	14.36–6.759	15.44–7.609	16.56–8.457	17.67–9.303
Average	− 18.29	− 16.99	− 15.69	− 14.39	− 13.10	− 11.81	− 10.51	− 9.27	− 8.29	− 7.44	− 6.81	− 6.92	− 7.44	− 8.06	− 8.79	− 9.87	− 11.04	− 12.22	− 13.41	− 14.62	− 15.82
SD	5.01	4.70	4.41	4.12	3.81	3.49	3.17	2.76	2.19	1.73	1.56	1.64	2.09	2.71	3.38	3.76	4.03	4.30	4.59	4.89	5.20
*Maximumm*																					
Rank	9.72–19.65	8.94–18.23	7.92–16.80	7.32–15.37	6.71–13.94	6.10–12.5	5.58–11.05	5.07–9.608	4.37–8.409	3.84–7.311	3.32–6.567	2.81–5.821	2.42–5.209	2.82–4.755	2.73–4.299	2.69–4.884	3.69–6.271	4.54–7.656	5.36–9.039	6.21–10.53	7.11–12.16
Average	14.01	12.77	11.54	10.52	9.58	8.67	7.77	6.89	6.07	5.36	4.77	4.28	3.85	3.60	3.44	3.88	5.10	6.35	7.61	8.87	10.13
SD	4.73	4.56	4.40	4.08	3.67	3.27	2.85	2.43	2.06	1.76	1.52	1.36	1.23	0.87	0.61	0.81	1.03	1.26	1.50	1.75	2.01

### Risk of atypical femoral fracture

**Fig. 5:**
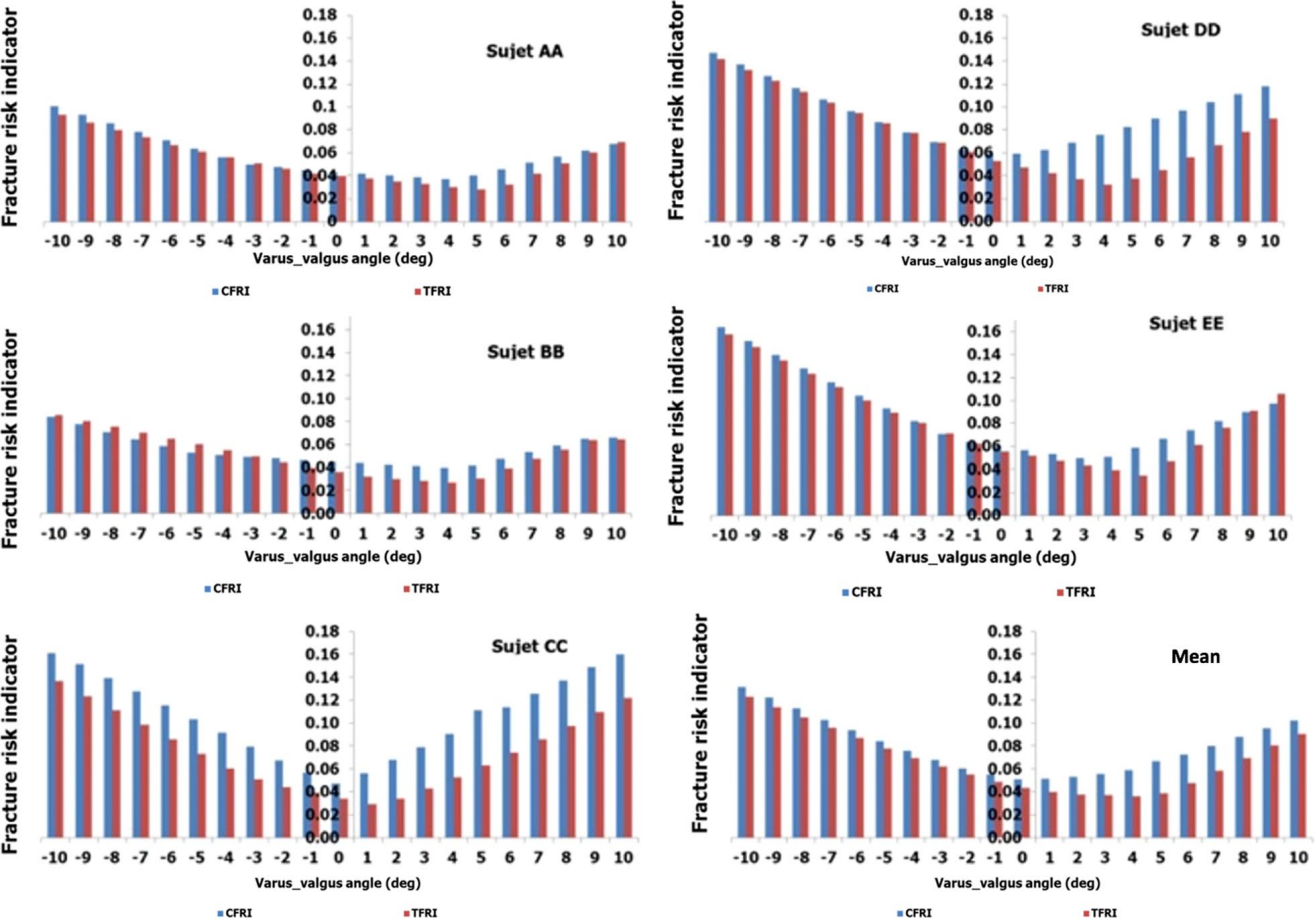
Femoral diaphysis fracture risk indicators for all subjects in all configurations

To study the effect of misalignment on the risk of femur fracture, fracture indicators and tensile and compressive stress limits for the proximal femur and diaphysis were calculated separately. In all cases, the FRI based on the main stress criterion were calculated for the femur diaphysis in standing position, taking into account the inclination or direction of the load. The calculated FRI are shown in Fig. 5.

Increased risk indicator of diaphysis fracture was observed for maximum varus and valgus deformations. The FRI fracture risk indicator of the femoral diaphysis varies proportionally with the absolute value of the steering angle (Table 4). However, the largest varus deformation simulated (− 10°) found a higher risk of diaphysis fracture indicator than in valgus (10°).

**Table 4:** Femoral diaphysis fracture risk indicator averages in varus/valgus for all five subjects

FRI risk indicator invoice																					
Angle(deg)	− 10	− 9	− 8	− 7	− 6	− 5	− 4	− 3	− 2	− 1	0	1	2	3	4	5	6	7	8	9	10
*Compressive*																					
Range	0.084–0.163	0.077–0.152	0.070–0.140	0.064–0.128	0.058–0.115	0.052–0.104	0.050–0.093	0.049–0.082	0.047–0.071	0.045–0.063	0.043–0.060	0.041–0.059	0.040–0.067	0.038–0.079	0.036–0.090	0.040–0.111	0.045–0.113	0.051–0.125	0.056–0.137	0.061–0.148	0.065–0.160
Average	0.131	0.122	0.112	0.103	0.093	0.084	0.075	0.067	0.06	0.054	0.051	0.051	0.053	0.055	0.058	0.066	0.072	0.08	0.087	0.095	0.101
SD	0.036	0.034	0.032	0.029	0.026	0.024	0.02	0.016	0.011	0.008	0.008	0.008	0.012	0.017	0.023	0.03	0.029	0.031	0.033	0.035	0.039
*Tensile*																					
Range	0.085–0.157	0.080–0.146	0.075–0.134	0.070–0.123	0.064–0.111	0.059–0.100	0.054–0.089	0.049–0.080	0.043–0.071	0.038–0.062	0.034–0.055	0.029–0.051	0.029–0.047	0.028–0.043	0.026–0.052	0.028–0.063	0.032–0.074	0.041–0.085	0.051–0.097	0.060–0.109	0.064–0.121
Average	0.122	0.113	0.104	0.095	0.086	0.077	0.069	0.061	0.054	0.048	0.043	0.039	0.037	0.036	0.036	0.038	0.047	0.058	0.069	0.080	0.090
SD	0.031	0.029	0.026	0.023	0.021	0.018	0.016	0.015	0.014	0.011	0.01	0.009	0.007	0.006	0.010	0.014	0.015	0.017	0.018	0.020	0.024

The decrease in varus from − 10° to 0° decreased the mean value of the compression fracture risk indicator and the mean value of the traction fracture risk indicator from 0.131 (SD = 0.036) to 0.051 (SD = 0.008) respectively, and from 0.122 (SD = 0.031) to 0.043 (SD = 0.010). The increase in valgus deformation from 0° to 10° increased the mean value of the compression fracture risk indicator and the mean value of the traction fracture risk indicator respectively from 0.051 (SD = 0.008) to 0.101 (SD = 0.039), and from 0.043 (SD = 0.010) to 0.090 (SD = 0.024).

## Discussion

Axial deformation in varus of the lower limb, all causes combined, presented a higher risk indicator of femoral diaphysis fracture than in valgus. This means that varus alignment could play a major role in AFF pathophysiology. The diaphyseal fracture risk indicators have the highest values for extreme axial deformation (-10°/10°). This shows a strong correlation between AFF risk and the mechanical axis in varus/valgus.

In the literature, some studies have associated diaphyseal femoral stresses with axial deformities due to femoral curvilinear (primary or secondary) malformations. Oh et al. [9] demonstrated using a tomography-based finite element method that patients with arched femoral diaphysis had greater stresses on the anterolateral surface of the diaphysis. Sasaki et al. [8] compared the femoral curvature of nine elderly patients treated for low energy diaphyseal femoral fractures with those of 24 controls without fractures. They reported that femoral curvature was significantly higher in patients with AFF compared to the control group, suggesting that an increase in femoral curvature could be a causal factor of AFF. For the same purpose, Morin et al. studied femoral geometric parameters using EOS imaging of 16 Caucasian women with AFF [20]. Their analysis showed that these patients had a more laterally curved femur of − 3.2° (SD = 3.4) versus − 0.8° (SD = 1.9) for the control group. Our study evaluated the femoral stress distribution as well as the risk of AFF exerted on a member in varus, whatever its origin: femoral, tibial, or femoral-tibial. The observed results highlight the importance of considering axial deformation as a whole, and not only on the curvilinear character of the femur.

Regarding the location of the fracture, for Oh et al. [9] they appeared to be full-blown fatigue fractures of the mid-shaft diaphyseal fractures on the curved femur. The location of the atypical fracture could then be determined by the individual distribution of the stresses related to the curvature of the femur as well as its cervical-diaphyseal angle [21]. Models with significant varus deformation had higher tensile stresses at the end of the distal lateral diaphysis of the femur, which may indeed explain the mid-shaft diaphyseal location of some AFF. The models with the least axial deformation had increased tensile stresses in the proximal region, which may explain the occurrence of sub-trochanteric fractures. These observations are consistent with the study by Saita et al. [21], which evaluated the alignment of the lower limbs in standing position in 10 patients with AFF [22].

They found that the mechanical axes of the lower limbs, represented by the femoral-tibial angle (FTA), correlated with fracture height. Patients with medial-diaphyseal AFF had larger FTA (183.3°), while patients with sub-trochanterian AFF had smaller FTA (172.8°), concluding that the alignment of the lower limb affected the location of the fracture. In addition, the femoral morphological parameters reported in AFF populations in the Morin study [19] presented a wider alignment of varus at the knee joint relative to the control group (− 1.6° (4.2) vs − 0.4° (1.9)). Haider et al. [10] also attempted to determine the most important morphological parameters for AFF. They found that the greatest variations were caused by the radius of the femoral diaphysis and the angle of lateral curvature [20].

Our results therefore reinforce the idea that the abnormal mechanical properties of the femoral diaphysis may be due to misalignment of the lower limb and are associated with the development of AFF. Impairment of bone tissue properties, classified as minor criteria by ASBMR [23, 24] should also be considered.

Prolonged alteration of bisphosphonate-induced bone remodeling [25] or suppression of bone remodeling would result in deterioration of bone microarchitecture, reduce the bone repair process, and result in accumulation of bone micro-damage, source of low-energy diaphyseal femoral fractures or AFF [23]. An accurate estimation of fracture risk is therefore required before introduction of bisphosphonate therapy in osteoporotic patients [7, 26, 27]. For a long time, AFF was considered in terms of transverse stress fractures occurring in the lateral femoral diaphysis associated with increased bone fragility, itself associated with long-term anti-resorption therapy [2]. Meng Ai Png et al. [28] showed that bisphosphonate treatment was associated with periosteal femoral stress reaction called radiographic “black line”. An increased risk of AFF was observed in lateral cortical periosteal response, especially in cases of painful symptomatology [25, 29]. The pathophysiology of AFF is not yet fully understood. The fracture profiles and their different locations show their multifactorial character. The combination of axial deformation stress and bone fragility create an environment conducive to the development of AFF.

The limitations of our FE modeling come from a small sample of patients whose original FTA we did not know about. Similarly, the applied boundary conditions did not take into account the specific weight of each subject. Stress distribution is directly related to the geometry and we have not varied the femoral geometry, even though variation can be the cause of the modification of the FTA, as is the case of curved femoral bone. Nor we did not consider the alteration of bone tissue in our model, but it remains a minor factor of AFF.

## Conclusions

Variations in the mechanical axis of the lower limb influence stress distribution at the femur diaphysis and increase the risk of AFF. The axial deformation in varus is particularly at risk of AFF, whatever its origin: femoral, tibial; or femoral tibial deformity. Although the fracture profiles and their different locations show their multifactorial character, varus deformation seems to be a determining factor. The combination of axial deformation stresses and bone fragility consequently contribute to the creation of an environment favorable to the development of AFF.

## Data Availability

Not applicable.
